# Using the nominal group technique to involve young people in an evidence synthesis which explored ‘risk’ in inpatient mental healthcare

**DOI:** 10.1186/s40900-017-0069-8

**Published:** 2017-09-01

**Authors:** Nicola Evans, Ben Hannigan, Steven Pryjmachuk, Elizabeth Gillen, Deborah Edwards, Mirella Longo, Gemma Trainor, Felicity Hathway

**Affiliations:** 1 School of Healthcare Sciences, Cardiff University, Heath Park, Cardiff, CF14 4LN UK; 2 Cardiff University, Cardiff, UK; 3 University of Manchester, Manchester, UK; 4 King’s College, London, UK; 5 University of West of England, Bristol, UK

**Keywords:** Nominal group technique, Young people, Mental health, Inpatient, Research involvement

## Abstract

**Plain language summary:**

We conducted a review of research on the topic of ‘risk’ in hospital based mental health care for young people aged 11-18. We wanted to include a contribution from young people alongside other stakeholders with expertise to guide the research team in decisions made setting parameters for the review. To achieve this, we held a stakeholder group meeting. We used the nominal group technique, a method designed to create a structure and a process for getting feedback from a group of people in a way that allows everyone to have an equal say. In this study, we show how our use of this approach enabled our stakeholder group to shape the focus of our study towards an area of more importance and relevance to them.

**Abstract:**

**Background:**

In this paper we demonstrate how our application of the nominal group technique was used as a method of involving young people with previous experience of using inpatient mental health services in an evidence synthesis.

**Methods:**

Nominal group technique is an approach to group decision-making that places weight on all participants having an equal opportunity to express a view, and to influence decisions which are made. It is an effective way to enable people who might otherwise be excluded from decision-making to contribute.

**Results:**

In this study, the focus of the evidence synthesis was significantly shaped following using the nominal group technique in our stakeholder advisory group meeting. The young people present in the group invited the research group to think differently about which ‘risks’ were important, to consider how young people conceptualised risk differently, focussing on risks with long term impact and quality of life implications, rather than immediate clinical risks.

**Conclusions:**

Using the nominal group technique with young people did offer a method of promoting the equality of decision making within a stakeholder advisory group to an evidence synthesis project, but care needs to be taken to invite sufficient young people to attend so they can be proportionally represented.

## Background

In this paper we offer an approach to involving young people in the course of conducting an evidence synthesis into the risks for young people admitted to inpatient mental healthcare. As a group of health service researchers we shared a common belief that research ‘with’ rather than ‘on’ people should be our aspiration [1]. We have used the National Institute for Health Research INVOLVE definition of involvement as ‘research being carried out ‘with’ or ‘by’ members of the public’. When designing our study we also were mindful of Article 12 of the United Nations Convention on the Rights of the Child (UNCRC) which clearly state that children and young people have a right to a say about all matters that affect them. Care provision within mental health services, and research into this field is included within that definition. To that end, we wanted to ensure that we could embrace the contributions of young people who had experience of inpatient mental healthcare in our study being mindful to reduce the potential impact of their perspectives being filtered by adults, a criticism of previous research with vulnerable children and young people [1]. One of the young people who was involved in this work contributed to the development of this paper by reviewing and editing an earlier draft.

We were mindful that there are a number of models to inform the involvement of young people in research or community projects, many of which are based on the Hart ladder of participation [2]. However, as our study was an evidence synthesis, rather than an empirical study, we had an obligation to consider what approach might be usefully employed to involve young people in a meaningful way in this specific context. There are evidence syntheses reported in the literature that have used different strategies to involve young people as part of their process, including an example of an Evidence for Policy and Practice Information and Co-ordinating Centre (EPPI-Centre) approach systematic review [3] and in a public heath systematic review looking at the effects of schools and school environment interventions on children and young people’s health [4]. In this paper we discuss how our application of the nominal group technique was used as a method of involving young people who had experience of using mental health services, and therefore had a degree of expertise about that care system through their experience. Throughout the paper, we refer to young people being involved in the project. We do not report on the gender or ages of these young people, but by definition child and adolescent mental health services in the UK (CAMHS) provides for children and young people aged eighteen or younger.

### The RiSC study: An overview

The RiSC study was funded by the National Institute for Health Research (NIHR) Health Services and Delivery Research (HS&DR) Programme to bring together research and other evidence in the area of ‘risk’ for young people in inpatient mental health care. A full, open access, report of the totality of the project has been published [5] along with a summary of main findings [6]. The study was designed in the knowledge that, typically, the term ‘risk’ used in the context of mental health services refers in a relatively narrow way to the chances of harm to self or others through suicide, self-injury or violence. In the RiSC project we purposefully adopted a broader understanding of the word, recognising ‘risk’ as multidimensional, complex and contextual. We recognised that risk-taking can be a route to resilience [7], and also that the experience of mental ill-health and admission to hospital presents risks to a young person’s maturation and development, and to his or her social networks, family relationships and educational progression.

In acknowledging the different ways in which ‘risk’ may be used, and of the differing interests and priorities of people with concerns in the CAMHS field, we were concerned to conduct our evidence synthesis in ways sensitive to stakeholder views and experiences. This led us to the two-phase EPPI-centre approach [8]. Like other traditions of evidence synthesis the EPPI-Centre approach encourages transparency and commitment to quality. Distinctively, it also stresses the importance of involving stakeholders at key points in the life of a project, giving people the opportunity to influence decisions and to shape a study’s overall direction. In a two-phase EPPI-Centre project a mapping, or scoping, of the overall area under review is followed by a targeted, in-depth, review and synthesis of the evidence in one or more sub-areas. A central role for stakeholders is in helping determine the focus of the second phase.

In phase 1 of the RiSC study we conducted a scoping review with the aim of mapping out research sitting at the intersection of four areas: ‘young people’, ‘mental health’, ‘inpatient’ and ‘risk’. In our interrogation of two bibliographic databases (MEDLINE and PsycINFO) we combined search terms reflecting these fields. In our theming of the content of the 124 individual items finally included we found an overwhelming majority concerned with clinical risks, such as the risks of suicide, harm to self and harm to others. Understanding, and addressing, these risks is a vitally important task in the inpatient CAMHS arena. However, reflecting our use of the EPPI-Centre approach it was at exactly this point that we suspended our searching and theming in order to consult with stakeholders able to guide the focus of the second, in-depth, phase of our project.

### Involving young people

The creation of a stakeholder advisory group for the study was a key stage within this evidence synthesis. Decisions on who to invite to the stakeholder advisory group were taken collectively by the project team, drawing on our understanding of the CAMHS system and our working relationships with the field. Four professionals accepted our invitation and attended. Aside from professionals working in the field, we wanted to ensure that the views of young people who had used CAMHS were also represented in this forum. One of our project team was from YoungMinds, which is a mental health charity for children and young person’s mental health in the UK. YoungMinds was involved with this project from the outset, during the preparation of the project’s outline proposal for funding. Following confirmation of this project’s funding award, a key meeting was convened with YoungMinds’ with a view to mapping the fine detail of how young people might most effectively be involved in the work of determining phase 2 priorities. YoungMinds was engaged in a piece of work to increase the opportunity for young people affected by mental health difficulties to share their views and experiences and to influence research in that area. It was agreed therefore, using part of our project funding, that YoungMinds would access young people from their existing networks to conduct a piece of work on the project team’s behalf. Five separate conversations were held with young people wo had previously been in CAMHS inpatient services. They were asked to discuss a series of questions we had formulated about ‘risk’ further to stage one of the EPPI-centre review detailed above. Their feedback was captured and anonymised and then presented to the stakeholder advisory group by the YoungMinds colleague. In addition two young people with experiences of using child and adolescent mental health services agreed to attend the stakeholder advisory group having been invited by one of the research team. Young people recruited by the clinician research team member were supported throughout [1, 9]. Although no parent was able to attend the stakeholder advisory group itself, one mother, unrelated to any of the young people involved, was able to send feedback through a project team member. So in the meeting itself were two young people, and feedback from a further five young people and one parent, all with experience of inpatient CAMHS. There was no determination in finding a specific number of people who might accurately represent the breadth and range of young people who access CAMHS in terms of factors such as age, gender, or geographical location. We were thankful that we were able to involve young people in this evidence synthesis to represent their own views. As this project was not a data-generating study, we were clear to distinguish this involvement of young people from the activity of directly participating in research and thus research ethics committee approval was not needed, (http://www.invo.org.uk/resource-centre/library-resource/?id=406§ion=involve).

## Methods

### Using the nominal group technique to the RiSC study

Nominal group technique is an approach to group decision-making introduced in 1975 by Delbecq and Van de Ven [10]. It places weight on all participants having an equal opportunity to express a view, and to influence decisions which are made [11]. It is an effective way to enable people who might otherwise be excluded from decision-making to contribute. For example, Tuffrey-Wijne et al. [12] show how nominal group methods can be used to involve people with learning disabilities. The approach can also be a useful strategy for eliciting views from stakeholders on priorities from a set of options, when trying to make decisions through a group process [13]. The accepted format for applying this technique is to use a five step format as described by Carney et al. [11] by firstly using an opening statement to set the scene, then enabling a silent generation of ideas, followed by an invitation to a ‘round robin’ of feedback, a clarification of ideas and finally a voting and ranking process of the ideas generated.

In the RiSC evidence synthesis, the nominal group technique was used as a strategy to generate the priorities for the *second phase* of the EPPI evidence synthesis. There now follows a stepwise description of how the nominal group technique approach was applied in this case.

**Fig. 1:**
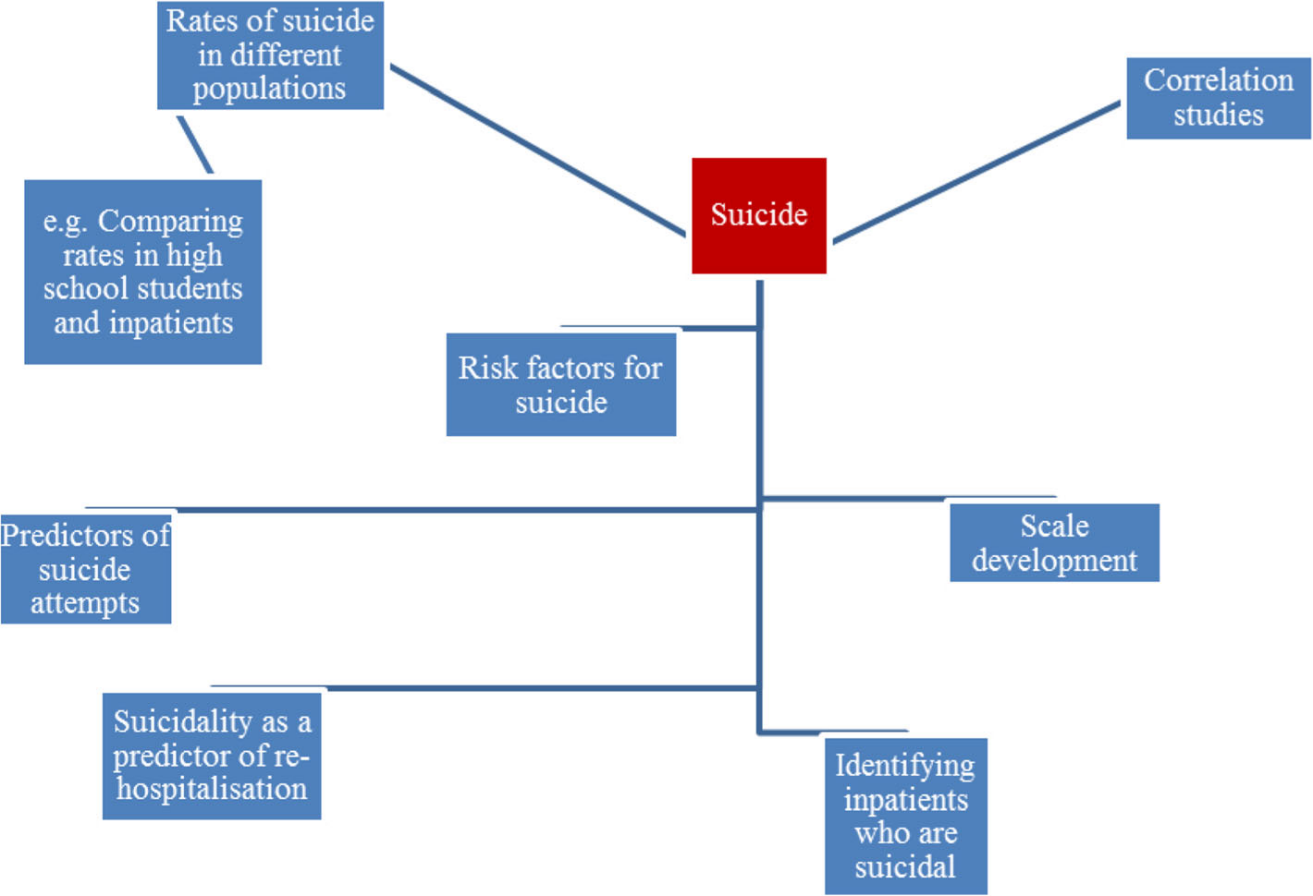
Table of ranked priorities for phase 2 for each person present (*n* = 14) which had been collated following a combined project team/stakeholder meeting

- Step 1. Opening statement: introduction and explanation
  An overview of the RiSC study was presented to the stakeholder advisory group, setting out the study aims and presenting the broad themes arising from the completed phase 1 scoping and mapping exercise. There were fourteen people present in the stakeholder advisory group meeting. This group consisted of members of the project team [*n* = 7], CAMHS managers [*n* = 1], CAMHS practitioners [*n* = 3] from different backgrounds, young people with experience of inpatient mental health services [*n* = 2], a key collaborator from Young-Minds who presented the views of young people [*n* = 5] with whom he had consulted. The views of a parent, previously consulted, were reported to the group. As an enduring record for participants to consult and review, we displayed around the meeting room flipchart (A1) size diagrams of the phase 1 identified themes. An example of a diagrammatic representation of these themes is given in Fig. 1.
  Accompanying this was a verbal presentation from our YoungMinds collaborator in which the key outcomes of the completed consultation previously conducted with young people were shared and also presented visually on a flipchart. Some feedback from a parent was also presented. Presenting this range of information in multiple formats allowed the stakeholders present the opportunity to have equal access to the information drawn from phase 1 of the evidence synthesis. This information provided the raw material for the stakeholder advisory group to assimilate and consider.
- Step 2. A silent generation of ideas
  In this step of the process, the stakeholders were invited to think about, and to record in writing on post-it notes, responses to the following question, ‘What do you think are the risks for young people as they make the transition into, through and out of inpatient CAMHS?’ This had direct relevance to the focus of the funded evidence synthesis. Stakeholders were invited to do this as individuals without discussion within the wider room. The rationale for not speaking during this process was to ensure each contributor could create and share their own thoughts without prematurely being influenced by comments from others. Written responses also allowed us to preserve a record for use following the meeting’s completion.
- Step 3. The sharing of ideas using a ‘round robin’ technique: exploring categories of risk for the in-depth review of evidence
  With a member of the project team acting as facilitator, all meeting participants were invited to individually share their written responses. These were recorded on A1 flipcharts using the words directly spoken. During this round the views of the parent who had previously been consulted were also recorded on a flipchart.
- Step 4. The clarification of ideas through a group discussion
  By this juncture, all those present had access to a large volume of spoken information, and written information displayed around the meeting room which included our first phase maps produced through our scoping and theming of the content of the 124 items located in our database searches, a summary of the views of young people that had been generated and presented by YoungMinds, some feedback from a previously consulted parent as well as individual ideas that had been collected from all those present.
  We then facilitated a whole-group discussion aimed at making sure that all ideas, views and experiences previously presented and displayed were understood by everyone. We also wanted to make sure that we had available for all participants a complete list of the full range of ‘risks’ associated with inpatient CAMHS derived from our mapping of previous research, and people’s ideas and experiences.
- Step 5. Voting and ranking
  In this final section of our meeting we asked all those present to rank, in writing, the order in which the risks identified should be prioritised for the purposes of our in-depth, second phase review and synthesis. Following the meeting’s end we collated these into a long list, which is reproduced in [Table 1] which contains an illustrative sample of the ordered priorities from this consultation.

**Table 1:** An illustrative sample of how three stakeholders ranked the order in which the risks identified should be prioritised for the purposes of phase 2 review and synthesis

1. Bad influences, unhelpful friendships, new peer norms
2. Imitating other patients
3. Attitudes of staff/therapists, dysfunctional systems, lack of staff training to deal with such behaviour
4. Early discharge/making decisions about discharge without consulting patient
5. Not acknowledging risk factors that are important to the young person’s wellbeing
6. Transition back into community/adult services. Not enough planning or coping strategies in place to help patient
7. Unable to engage with staff members/being isolated increases bad feelings, disempowerment of family members
1. Self-injury
2. Suicide
3. Disempowerment/distress/no responsibility towards self, no motivation to recover
4. Violence from patients
5. Unhelpful friendship with other young people on the unit
6. Having a new network of friends that all have mental health problems
7. Contagion: learning and emulating behaviours witnessed in other patients
8. Risk to education
9. Stigma on return to mainstream education
10. Unmonitored visitors taking advantage of young people’s vulnerability (abuse)
1. Losing ‘normal’ identity
2. Not having the chance to achieve educationally
3. Risk adverse culture leads to lack of progress and increased readmission
4. Risk of losing connection to social group leads to a longer recovery time

## Results

From this complete list we extracted the top three priorities from each of the 15 cells, to produce a shorter list of 45 individual items. To group these into broader categories this list was coded and themed, without conferring in the first instance, by two members of the project team. Their joint categorisation was checked by a third member of the team. By the end of this process we had produced a prioritised list of eight categories of risk, each having a count of the number of items from the total of 45 subsumed within it. As part of this process we subsumed a number of items within the categories ‘dislocation’ and ‘contagion’, which we defined as below.

- Dislocation includes the ideas of young people being removed from ‘normal’ life, of being ‘different’ and experiencing stigma or discrimination. Dislocation also captures the ideas of young people losing their previous identities, their existing social contacts and friendships groups. It includes isolation from educational settings and opportunities for social contact. In summary, ‘dislocation’ implies an unhelpful loss.
- Contagion implies an unhelpful gain in our study, such as learning about and acquiring more problematic behaviour and the formation of new and unhealthy friendships.

These priority categories, with item counts, are displayed in Table 2.

**Table:** Table 2

Area of risk	No of times identified
Dislocation	16
Contagion	6
Harm from organisation	6
Institutionalisation	5
Self-harm	4
Decision-making	3
Suicide	2
Aggression	1
Other (managing dissonance/ambivalence (*n* = 1) and psychological risks (*n* = 1))	2

We included this information in a post-meeting document circulated to stakeholders, along with an explanation of our new categories of ‘dislocation’ and ‘contagion’ and an invitation to all those who had contributed to the stakeholder advisory group either in person or through representation for any final observations before we initiated the in-depth second phase of the study. This concluded the activity for that stakeholder advisory group meeting and the use of the nominal group technique. There was a further stakeholder advisory group meeting towards the end of the project to discuss dissemination processes. Examination and analysis of the ranked priorities identified through this process enabled a clear focus for phase two of the evidence synthesis, with an emphasis on ‘less obvious risks’ such as education, family and peer relationships and a move away from clinical risk such as self-harm and aggression as identified by the stakeholders during this process.

## Discussion

One of the challenges posing us as a research team was how to involve young people in a meaningful way in an evidence synthesis. There has been some criticism about the lack of actual involvement of young people in health research affecting them. When looking at the literature to determine how best to do this, we found that either young people’s views were represented by their parents or carers, or where young people had been involved directly, the detail of this was difficult to determine [14]. Whereas our approach did not offer a new methodology per se to facilitate effective participation, it did demonstrate how using the nominal group technique might be useful in engaging with young people in an evidence synthesis. We hope that our experience in this study will encourage a wider involvement of young people into evidence syntheses. There are limitations in the way the nominal group technique has been used in this case. Should we conduct a future evidence synthesis with young people, we would consider inviting a young person to lead the advisory group, with appropriate training in nominal skills technique (if required) and preparation for the young person beforehand.

Using the EPPI approach to evidence syntheses allowed for a two-step approach and thus, by design, created a stage within the review process for a pre-determined opportunity for stakeholder involvement. We wanted to create a forum in which all stakeholders had a sense of being equal and their contribution equally valuable to the decision making process in this review. In the DePICTED empirical study [15], the research team became aware that there were limitations of including young people in the same stakeholder group as adults, particularly parents, and believed that this combination might have a counter-productive effect by hindering the young people from articulating their views. Consequently they separated the stakeholders into adults/practitioners/parents and young people to promote ‘active engagement…to [enable participants to] step outside their usual generational roles, and to disclose information that they might not wish to share with their parents’ [15]. The method of organising the generation of feedback within each of the stakeholder groups was to use the nominal group technique approach and a discrete choice experiment to prioritise aspects of their study. In a difference to Lowes et al.’s [15] work, we found that we were able to facilitate the young people’s expression in the stakeholder group through a small number of complementary strategies. YoungMinds was able to represent the views of young people ‘outside of the room’ through a prior consultation exercise, and the young people in the room were supported by an accompanying practitioner (GT). The activity of using post-it notes in step 2 (‘silent generation of ideas’) of the nominal group technique enabled individual contributions to be made without any potential social discomfort of speaking aloud in a meeting with unfamiliar people present. One of the weaknesses in our study was that were only two young people present in the actual stakeholder advisory group. A stronger young person’s voice might have been achieved through the physical presence of more young people, possibly with a young person chairing the consultation. Although there was a meeting held towards the end of the project to discuss dissemination processes, having more frequent stakeholder advisory group meetings along the process of the evidence synthesis would have ensured a more robust strategy for involving young people.

In the RiSC evidence synthesis, there was a significant impact on the direction of travel of the evidence synthesis between the two stages of the EPPI-Centre review following the stakeholder advisory group in which the nominal group technique was used. The young people invited the research group to think quite differently about the ‘risks’ to their peers during the transition through inpatient care, and to concentrate on those aspects that had a longer term impact reflecting quality of life aspects. Specifically they asked us to consider the impact that an inpatient stay might have on young people’s education, friendships and relationships with family members following a period of extended admission to an inpatient facility. The young people with whom we collaborated on this study also raised the important issue of how stigmatising one might find a period of inpatient mental health care. Given most of the literature we had retrieved in stage 1 of the EPPI review of evidence had concentrated on the topics of suicide, self-harm and aggression, those areas that frequently challenge clinicians, the young people and parent collaborating on the RiSC study invited a new perspective on the issues that have importance for people using mental health inpatient services for young people. When we think about the influence on the study direction from this intervention, and map it onto the ladder of children’s participation [2], our responding to the clear feedback of the young people who had experience of inpatient mental healthcare resonates with the higher aspirational levels of children’s involvement and sits within the category ‘adult-initiated, shared decisions with children (or, in our case, young people). McLaughlin [16] has suggested that young people are inherently vulnerable in the research arena and it is often difficult for their views to be heard on the same level as adults given the power imbalances that exist, but using the nominal group technique in this case did appear to influence the decisions that we made about the focus of stage 2 of this EPPI-centre evidence synthesis and thus in part helped to counteract any apparent power imbalance in the stakeholder advisory group meeting.

Reflecting on the process of using the nominal group technique with young people, we recognise that there were technical aspects of this that might benefit from further adaptations if being used with children or young people in the future. In the RiSC example, no icebreaking or warm up exercises were used to encourage contribution from young people [1, 17]. This omission may have impacted upon the confidence that the young people felt in voicing their ideas within a group that was largely populated by adults who were not familiar to them. Particularly if inviting young people or children, brief fun activities that involve moving around the room, drawing on flip charts and post-it notes might help engagement in the process. In empirical research with children and young people, there are suggestions that creative and participatory methods, such as drawing, are a useful method to aid articulation of ideas and to involve children and young people [18, 19]. However, in our view we need to be clear that *participating* in research as described by Mannay [18] and Punch [19] is different from *collaborating* or *being involved* in research. It requires a different response, a different role in relation to the study and thus arguably different techniques or approaches to facilitating. In this evidence synthesis, it was clear to us as the research team that the young people had influenced the direction and focus of the second part of our evidence review, but it was not until sometime later that one of the participating young people had a true appreciation of the impact of their involvement.

‘*As a young person involved with the project, it has been empowering to witness how our contribution significantly altered the direction of the research. The evolution of this project was considerably influenced by coproducing with young people in a way that extended beyond tokenistic participation and my hope is that this resulted in a more authentic reflection of how risk was conceptualised by young people whilst highlighting some of the disparities that exist*.’

The delay of feedback for the young person highlighted how important it is to include participants in the summarising and final report writing prior to research dissemination activities so a timely acknowledgment of the value of the young people’s involvement can be felt. The findings from this evidence synthesis has triggered a strand of inquiry about the lack of evidence what helps to address the issues of ‘risk’ for young people during inpatient mental healthcare. The young people involved in this evidence synthesis were invited to join the research team in an initial brainstorming event, to decide upon a focus, consider research aims and a research plan for an ongoing study. We recognised that the involvement of young people in the evidence synthesis was pivotal and that using the nominal group technique was one method of attempting to ensure a fair and equal contribution was heard within the context of a stakeholder advisory group with a mixed membership. Our plan for the follow-on study has begun with involving young people from the outset, to create together the focus for further research.

## Conclusion

The RISC study was strengthened by the active involvement of a stakeholder advisory that contained representation from young people who had used inpatient mental health services. Their contribution significantly influenced the direction of travel on this project, by alerting us as the research team to the issues that were of relevance to them. The use of the nominal group technique did create an opportunity to promote equality in decision making in the stakeholder advisory group, but care needs to be taken to invite sufficient young people to attend so they can be proportionally represented.

## Abbreviations

CAMHS: Child and adolescent mental health services
EPPI-centre: Evidence for Policy and Practice Information and Co-ordinating Centre

## References

[1] Pinfold V, Szymczynska P, Hamilton S, Peacocke R, Dean S, Clewett N, Manthorpe J, Larsen L (2015). Co-production in mental health research: reflections from the people study. Ment Health Rev J.

[2] Shaw C, Brady L, Davey C (2011). Guidelines for research with children and young people.

[3] Hart RA (1992). Children’s participation from tokenism to citizenship.

[4] Rees R, Oliver K, Woodman J, Thomas J (2009). Children’s views about obesity, body size, shape and weight: a systematic review.

[5] Jamal F, Langford R, Daniel P, Thomas J, Harden A, Bonell C (2014). Consulting with young people to inform systematic reviews: an example from a review on the effects of schools on health. Health Expect.

[6] Hannigan B, Edwards D, Evans N, Gillen E, Longo M, Pryjmachuk S, Trainor G. An evidence synthesis of risk identification, assessment and management for young people using tier 4 inpatient child and adolescent mental health services. Health Serv Delivery Res. 2015; doi:10.3310/hsdr03220. 25996025

[7] Edwards D, Hannigan B, Gillen E, Longo M, Prymachuk S, Trainor G, Evans N. What do we know about the risks for young people moving into, through and out of inpatient mental health care? Findings from an evidence synthesis. Child Adolesc Psychiatry Ment Health. 2015; doi:10.1186/s13034-015-0087-y. 10.1186/s13034-015-0087-y PMC4689041 26702297

[8] Coleman J, Hagell A (2007). Adolescence, risk and resilience: against the odds.

[9] EPPI-Centre (2010). EPPI-Centre methods for conducting systematic reviews.

[10] PEAR (2010). Young people in research: how to involve us. Guidance for researchers from the PEAR young people’s public health group.

[11] Delbecq A, Van de Ven A (1975). Group techniques for program planning a guide to nominal group and delphi processes.

[12] Carney O, McIntosh J, Worth A (1996). The use of the nominal group technique in research with community nurses. J Adv Nurs.

[13] Tuffrey-Wijne I, Bernal J, Butler G, Hollins S, Curfs L (2007). Using nominal group technique to investigate views of people with intellectual disabilities on end-of-life care provision. J Adv Nurs.

[14] Harvey N, Holmes CA (2012). Nominal group technique: an effective method for obtaining group consensus. Int J Nurs Prac.

[15] Mawn L, Welsh P, Kirkpatrick L, Webster LAD, Stain HJ (2015). Getting it right! Enhancing youth involvement in mental health research. Health Expect.

[16] Lowes L, Robling M, Bennert K, Crawley C, Hambly H, Hawthorne K, Gregory JW (2010). DEPICTED study team. Involving lay and professional stakeholders in the development of a research intervention for the DePICTED study. Health Expect.

[17] McLaughlin H, McLaughlin H (2015). Involving children and young people in policy, practice and research: an introduction. Involving children and young people in policy, practice and research.

[18] Street C, McLaughlin H (2015). Children and young people as researcher and research advisors: perspectives from the NCB research Centre. Involving children and young people in policy, practice and research.

[19] Mannay D (2016). Visual, narrative and creative research methods: application, reflection and ethics.

